# Novel Antiproliferative Biphenyl Nicotinamide: NMR Metabolomic Study of its Effect on the MCF-7 Cell in Comparison with Cisplatin and Vinblastine

**DOI:** 10.3390/molecules25153502

**Published:** 2020-07-31

**Authors:** Laura Del Coco, Maria Majellaro, Angelina Boccarelli, Saverio Cellamare, Cosimo Damiano Altomare, Francesco Paolo Fanizzi

**Affiliations:** 1 Department of Biological and Environmental Sciences and Technologies, University of Salento, Prov.le Lecce-Monteroni, I-73100 Lecce, Italy; laura.delcoco@unisalento.it; 2 Department of Pharmacy–Pharmaceutical Sciences, University of Bari, Via Orabona 4, 70125 Bari, Italy; ma.majellaro@usc.es (M.M.); saverio.cellamare@uniba.it (S.C.); cosimodamiano.altomare@uniba.it (C.D.A.); 3 Department of Organic Chemistry, Faculty of Pharmacy, Center for Research in Biological Chemistry and Molecular Materials (CiQUS), University of Santiago de Compostela, 15782 Santiago de Compostela, Spain; 4 Department of Biomedical Sciences and Human Oncology, University of Bari, Piazza Giulio Cesare 11, 70124 Bari, Italy

**Keywords:** biaryl nicotinamide, breast cancer, antiproliferative activity, metabolomics, NMR spectroscopy, MVA

## Abstract

A ^1^H-NMR-based metabolomic study was performed on MCF-7 cell lines treated with a novel nicotinamide derivative (DT-8) in comparison with two drugs characterized by a well-established mechanism of action, namely the DNA-metalating drug cisplatin (cis-diamminedichloridoplatinum(II), CDDP) and the antimitotic drug vinblastine (vinblastine, VIN). The effects of the three compounds, each one at the concentration corresponding to the IC_50_ value, were investigated, with respect to the controls (K), by the ^1^H-NMR of cells lysates and multivariate analysis (MVA) of the spectroscopic data. Relevant differences were found in the metabolic profiles of the different treatments with respect to the controls. A large overlap of the metabolic profiles in DT-8 vs. K and VIN vs. K suggests a similar biological response and mechanism of action, significantly diverse with respect to CDDP. On the other hand, DT8 seems to act by disorganizing the mitotic spindle and ultimately blocking the cell division, through a mechanism implying methionine depletion and/or *S-*adenosylmethionine (SAM) limitation.

## 1. Introduction

**Figure 1:**
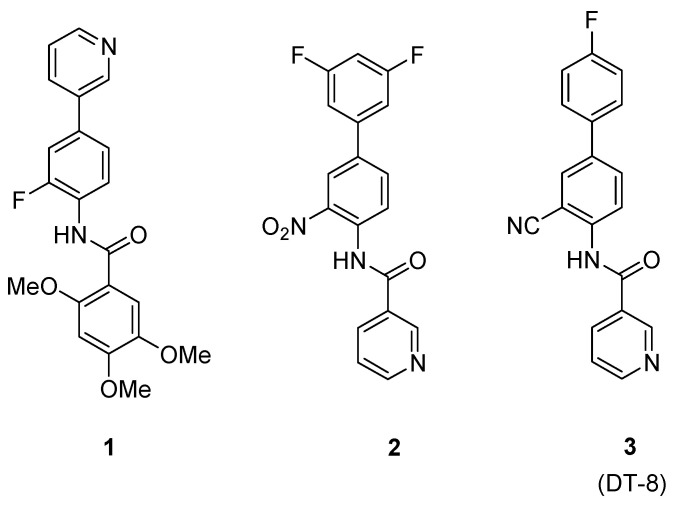
Structure of the hit 2,4,5-trimethoxybenzanilide **1** leading to the nicotinamide derivatives **2** and **3** (lab code: DT-8), the latter acting as in vitro potent nanomolar antiproliferative agent in the human breast cancer MCF-7 and MDA-MB231 cell lines.

The Nuclear Magnetic Resonance (NMR)-based metabolomic profiling of tumor cell lines, as well as of tissue samples, fine-needle aspirates and biofluids (e.g., serum, urine), has become one of the most promising diagnostic approach among the –omics techniques. This is not only to aid the staging of cancer, therapy monitoring, and hence personalized medicine, but also to provide a useful support for the identification of new therapeutic targets and the discovery of new anticancer drugs [1]. The wide diffusion of cancer disease and the continuous emergence of therapy-resistant tumors stimulates the endless search for new biomarkers, biomolecular targets, abnormal biochemical pathways and ultimately new treatment strategies [2,3]. To this regard, besides the combination therapies, the development of systematic methods for metabolomics is a promising strategy and tool for improving diagnosis and treatment [4,5]. In recent years, some of us investigated the potential anti-proliferative activity of an in-house synthesized library of diverse *N*-biaryl amides, including trimethoxybenzamides (e.g., **1**, Figure 1) [6,7] and nicotinamides (e.g., **2** and **3**, Figure 1) [8,9], with some of them showing very high antiproliferative activity against human breast cancer cell lines MCF-7 and MDA-MB231 [8]. The 2,4,5-trimethoxybenzanilide derivative **1**, which proved to be a potent P-glycoprotein (P-gp) inhibitor [6], was taken as the hit compound for an optimization study aimed at disclosing new chemical entities active toward P-gp-mediated multidrug resistant tumor cells. A number of replacements were designed for improving pharmacokinetics-related properties (e.g., water solubility) while retaining good antiproliferative effect against breast cancer, which is a major cause of women’s morbidity and mortality [2]. Among the synthesized compounds, the nicotinamide derivative 2 proved to inhibit the growth of MCF-7 and MDA-MB231 cell lines with IC50′s in the nanomolar range (250 and 229 nM, respectively) and tubulin polymerization at 3 μM concentration [6], whereas the bioisosteric replacement of NO_2_ with the nitrile CN group led to the nicotinamide derivative **3** (lab code DT-8) showing IC_50_ values equal to 47 and 37 nM on MCF-7 and MDA-MB231 cells, respectively [8].

From previous studies [8], DT-8 and VIN, used as a reference drug, revealed a similar dose-dependent accumulation of cells in the G2 and M phases and a decrease in the S phase, in the MCF-7cell cycle. This result suggested a similar mechanism of action, but DT-8 induced a G1 arrest at low doses, unlike VIN. Additionally, G1/G2 and M arrests were associated with reduced cell numbers, along with an increase in the sub-G1 population, indicative of apoptosis. Furthermore, DT-8 did not show similar effects on tubulin polymerization. These latest results indicated that DT-8 could act with a mechanism other than that of VIN. In fact, it was hypothesized that the high anti-proliferative activity of DT-8 could be associated with a multi-target mechanism [8]. With this background, we decided to study the effects of DT-8, VIN and CDDP using ^1^H-NMR-based metabolomics. CDDP was used, as an additional reference drug in order to clarify the DT-8 action also by comparison with possible known drugs with alternative targets (DNA) [10] and defined mechanisms.

To better understand the effect of the nicotinamide derivative **3** (DT-8) on the breast cancer metabolism, herein we carried out a ^1^H-NMR-based metabolomic study of the MCF-7 cell lines treated with DT-8 and two drugs with a well-established mechanism of action, namely the DNA-metalating drug cisplatin (CDDP) and the antimitotic drug vinblastine (VIN). The study of cancer-associated metabolic changes should help to shed light on the mechanism of action of anticancer drugs [11]. Among analytical tools, NMR spectroscopy and MS spectrometry, having their own advantages and limitations, are the most powerful tools for metabolomic profiling [12]. Some of us previously reported a ^1^H-NMR metabolic study of [Pt(O,O′-acac)(γ-acac)(DMS)], Ptac2S, in cisplatin resistant Epithelial Ovarian Carcinoma (EOC) to support the hypothesis that, even though they are both Pt (II) compounds, Ptac2S shows a totally different mechanism of action from CDDP, thus overcoming the drug resistance induction against cisplatin [8,9,10,11,13,14]. Herein, the effects of DT-8 (**3**) compared with CDDP and VIN on metabolic profiles of MCF-7 lysates, derived from cells incubated with the three compounds, each one at the concentration corresponding to the IC_50_ value, were investigated by an ^1^H-NMR-based metabolomic approach.

## 2. Results

### 2.1. H-NMR Analysis of Cell Lysate

**Figure 2:**
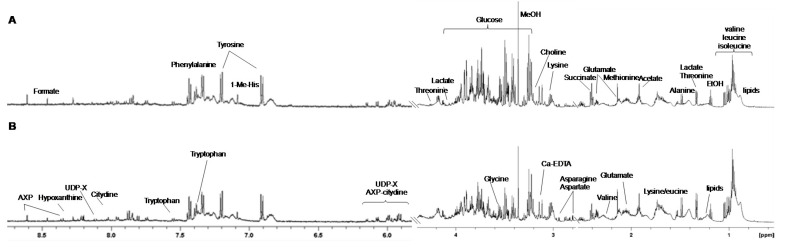
Representative one-dimensional 600 MHz ^1^H-NMR spectra (ZGCPPR) of lysate from the treated (**A**) and control (**B**) samples in the aromatic (left) and aliphatic (right) regions with some of the identified metabolites (AXP: adenosine mono, di-, triphosphate; 1-Me-His: 1-methyl-histidine; UDP-X: uridine diphosphate-glucose).

Representative NMR spectra from the controls (K) and treated samples with the identified metabolites (a total of 25, reported in Table S1) are shown in Figure 2. 2D ^1^H-^1^H COSY Correlation Spectroscopy, ^1^H-^13^C-HSQC Heteronuclear Single Quantum Correlation, and ^1^H-^13^C-HMBC Heteronuclear Multiple Bond Correlation and J-resolved spectra experiments were used to accurately identify and assign the metabolites, according to literature data [15,16,17,18]. The lysate spectra contained high-intensity signals from small molecules, such as formate, trypthophan, phenylalanine, tyrosine, α-β glucose, lactate, arginine, proline, aspartate, asparagine, alanine, glutamate, glutamine, methionine, acetate, succinate, valine, leucine, isoleucine, lipids, hypoxanthine and nucleotides (nitrogen-basis), such as adenosine-5′-monophosphate (AMP), cytidine, uridine-diphosphate (UDP)-glucose.

### 2.2. Multivariate Analysis of NMR Data

**Figure 3:**
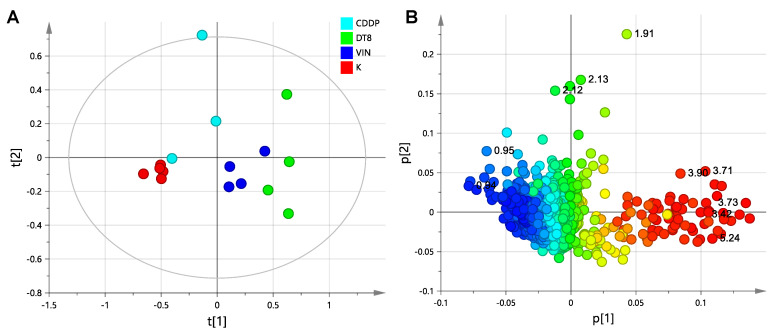
Principal component analysis, PCA score plot (**A**) for the whole data and corresponding color-coded coefficient loading plot (**B**) derived from the ^1^H-NMR spectra of cell lysate obtained from different groups. K: controls; CDDP: cis-platin, DT8: DT-8 treatment, VIN: vinblastine. The outer ellipse represents the 95% confidence interval (T^2^ Hotelling). Variables are the loadings, colored according to the absolute value of the correlation loading, p [1].

An unsupervised analysis (PCA, principal component analysis) was preliminarily applied to the whole data (ZGCPPR experiments), in order to investigate on the differences among samples, after the preprocessing of the NMR spectra (Figure 3A). The obtained PCA model (R^2^X = 0.71, Q^2^ = 0.41) revealed a marked groupings of controls (K) and a moderate clustering for VIN and DT-8 treated samples, with a good fit and classification of the model on the first principal component (t [1]), this explaining 55% of the total variance. By the color-coded coefficient loadings (Figure 3B and ^1^H chemical shifts reported in Table S1), valuable variable separation between the controls and treated samples were observed. In particular, a relative higher level of sugars (i.e., alpha and beta glucose) and a relative lower level of lipids were generally identified in all treated vs. control samples (K). It should be noted that one of the CDDP group (sample CDDP-3, being slightly outside of the 95% confidence interval, T^2^ Hotelling) was characterized by a relative higher level of N-acetyl residues, mainly responsible for the CDDP spread along the second principal component t [2].

**Figure 4:**
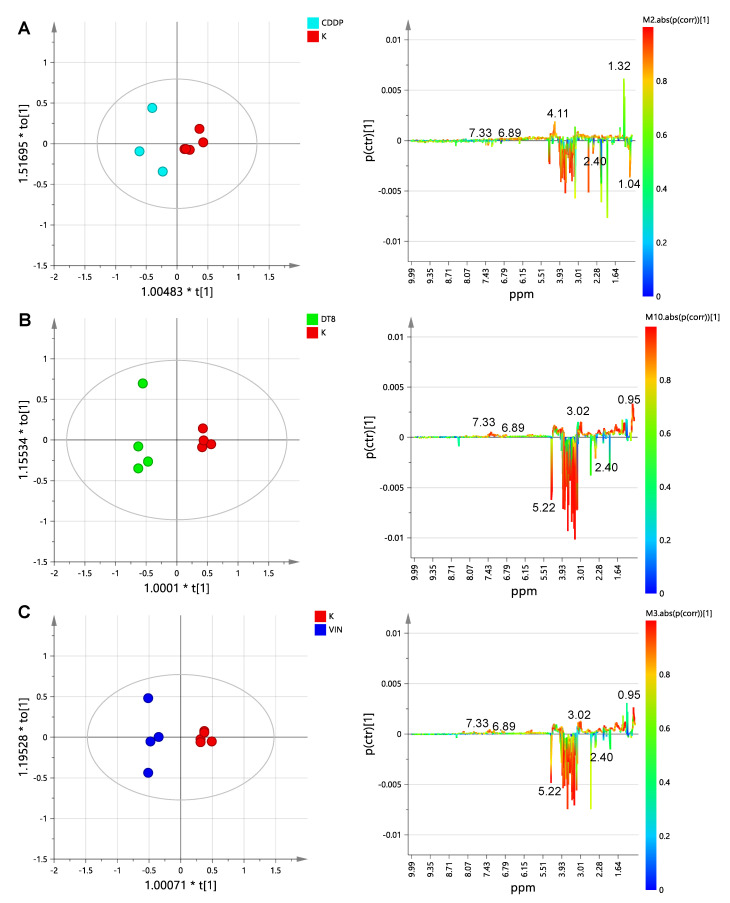
t[1]/to[1] orthogonal partial least squares discriminant analysis (OPLS-DA) score scatter plots (right) for (**A**) K vs. CDDP (**B**) K vs. DT-8 and (**C**) K vs. VIN cell lysates and the corresponding S-line plots (left) displaying the discriminant metabolites (the corresponding chemical shifts are reported in Table S1) and the related predictive loadings (Variables obtained from the ^1^H-NMR spectra are colored according to the correlation scaled loading (p (corr)).

The supervised orthogonal partial least squares discriminant analysis, OPLS-DA models were used to elucidate the most reliable class-discriminating variables that were highly diagnostic for controls (K) and the three treated groups. A pairwise comparison by OPLS-DA analyses was performed between controls (K) and treated samples (CDDP, DT-8, VIN). In particular, three different OPLS-DA models were obtained (Figure 4A–C) for K vs. CDDP, K vs. DT-8 and K vs. VIN samples, using one predictive and one orthogonal components. Model validation was performed by a permutation test (*n* = 20) and 7-fold internal validation. The Q^2^-intercept values from permutation tests were calculated to avoid the risk of over-fitting (reported in Figure S2 and Table S2). Statistical parameters of OPLS-DA models for classification were evaluated by comparing their Q^2^ predictivity (Table 1). In all cases, a good separation of treated samples, with respect to control samples, resulted from the analyses. In particular, the highest predictive ability (Q^2^ value of 0.979) was found for the K vs. DT-8 OPLS-DA model, followed by the K vs. VIN OPLS-DA model (Q^2^ = 0.905), while the K vs. CDDP model had the lowest predictive ability (Q^2^ = 0.684). It should be noted that a higher predictive ability of the model also indicates a higher differentiation of the sample classes. Therefore, as expected, the lowest differentiation with respect to the controls is observed for the CDDP-resistant cell strain (MCF-7) lysates. On the other hand, a higher difference is observed for the DT-8- and VIN-treated samples with respect to the controls. However, it should also be noted that a reasonable spread among treated samples (with respect to controls) was evident in all the three cases along the orthogonal component to [1], with the greatest value of R^2^Y for the OPLS-DA obtained for K vs. DT-8 samples (R^2^Y = 0.987), followed by the OPLS-DA of K vs. VIN R^2^Y = 0.976 and K vs. CDDP samples, with the last having R^2^Y = 0.85.

**Figure 5:**
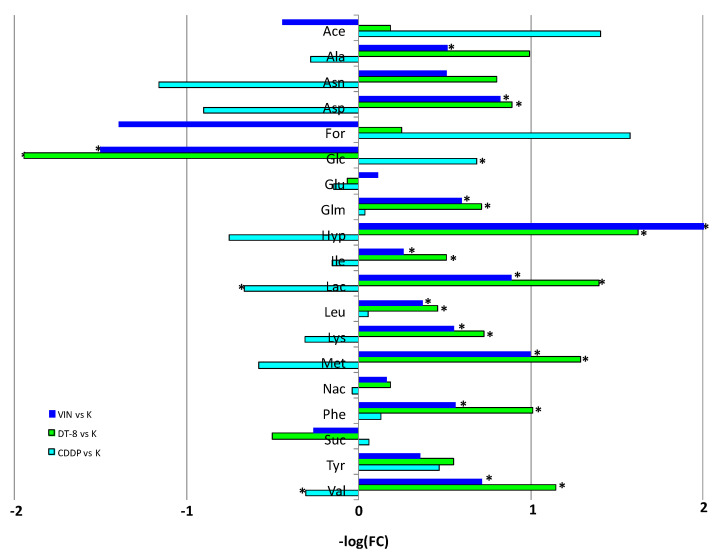
Discriminant metabolite comparison between treated samples (A: CDDP, B: DT-8 and C: VIN) and the controls (K). * The values of metabolites having Log_2_(FC) with *p*-value < 0.05 (x axis). (Ace: acetate; Ala: alanine; Asn: asparagine; Asp: aspartate; For: formate; Glc: glucose; Glu: glutamate; Glm: glutamine; Hyp: hypoxanthine; Ile: isoleucine; Lac: lactate; Leu: leucine; Lys: lysine; Met: methionine; Nac: *N*-acetyl moieties; Phe: phenylalanine; Suc: succinate; Tyr: tyrosine; Val: valine).

The discriminant metabolite comparison between treated samples (CDDP, DT-8, VIN) and controls (K) was obtained by studying the S-line plots and considering the FC ratio with *p*-value < 0.05 (reported in Table 2). A general relative higher content of glucose and a lower amount of lipids were observed in DT-8- and VIN-treated samples. Moreover, among the three types of treatments, DT-8 revealed the most significant metabolite variation, with relative low levels of aromatic aminoacids (phenylalanine, tyrosine), nucleosides, aspartate, asparagine, lysine, methionine, glutamate and alanine. The values of metabolites having significant Log_2_(FC) (with *p*-value < 0.05 x axis) are reported (Figure 5).

### 2.3. Evaluation of the Differences in Metabolic Profiles in MCF-7 Cells Obtained after Treatment with DT-8, VIN and CDDP

The metabolic map can serve as a tool for building hypotheses on metabolic processes in breast cancer and help develop strategies for therapeutic targeting of metabolism in cancer cells. To possibly obtain more detailed information on the mechanism of action of DT-8, the metabolic profiles of untreated MCF-7 cells were compared with those treated with DT-8, VIN and CDDP in the same experimental conditions (IC_50_ doses and 72 h of continuous incubation), and the evaluation of the data obtained from the ^1^H-NMR spectra acquired from cell extracts was performed (five controls K, four treated with DT-8 and VIN and three with CDDP). The different metabolite profiles (discriminant metabolites) obtained between samples treated with DT-8, VIN and CDDP showed a marked response between DT-8 vs. K and VIN vs. K with respect to the treatment with CDDP vs. K. This analysis of discriminating metabolites between treated samples (DT-8, VIN, CDDP) and controls (K) was made by studying both the S-line plots obtained from the OPLS-DA models and considering the related FC ratio with *p*-value < 0.05 (Table 2).

The comparison of the discriminating metabolites between treated and control samples (K) (Figure 5) identified a total of 19 metabolites. In particular, for treated DT8 vs. K samples, an increment of glucose and succinate, and a decrement for the other 17 metabolites (alanine, acetate, asparagine, aspartate, formate, glutamate, glutamine, hypoxanthine, isoleucine, lactate, leucine, lysine, methionine, *N*-acetyl moieties of glycoproteins, phenylalanine, tyrosine and valine) was observed.

The VIN vs. K analysis revealed a relative higher content for acetate, formate, glucose and succinate and a significant decrease for alanine, aspartate, glutamine, hypoxanthine, isoleucine, lactate, leucine, lysine, methionine, phenylalanine and valine.

Finally, the discriminant comparison of the metabolites between the CDDP-treated samples vs. K, identified significantly increased levels of lactate and valine (and not statistically significant, but also levels of alanine, aspartate, asparagine, glutamine, hypoxanthine, isoleucine, lysine and methionine) and a significant decrease in glucose (and not statistically significant, but also levels of acetate, formate and tyrosine). Moreover, as shown in Table 1, the highest predictive ability (Q^2^ = 0.98) was found for the OPLS-DA model obtained for K vs. DT-8, followed by the OPLS-DA model of K vs. VIN (Q^2^ = 0.90), whereas, in contrast, the K vs. CDDP model showed the lowest predictive ability (Q^2^ = 0.68).

Overall, the analysis of the samples treated with DT-8 and VIN (vs K) resulted in a significantly higher number of discriminating metabolites than those identified in CDDP vs. K. In the DT-8 vs. K and VIN vs. K profiles, the highest FC value resulted for glucose (FC = 3.85 and 2.84, respectively) and the succinate (FC = 1.42 and 1.20, respectively). The profiles of DT-8 vs. K and VIN vs. K also overlap each other for leucine (FC = 0.73 and 0.77, respectively) and alanine (FC = 0.50 and 0.70, respectively). In DT-8 and VIN vs. K, isoleucine and leucine content (FC = 0.70 and 0.73 for DT8 and 0.83 and 0.77 for VIN, respectively) is observed among the metabolites present in less quantities.

Interestingly, both DT-8 vs. K and VIN vs. K treated cells revealed a significant relative low content of hypoxanthine (FC = 0.32 and 0.15, for DT-8 and VIN, respectively), asparagine (FC = 0.57 and 0.70 for DT-8 and VIN, respectively), aspartate (FC = 0.54 and 0.57 for DT-8 and VIN, respectively) and methionine (FC = 0.41 and 0.50 for DT-8 and VIN, respectively). The remaining metabolites revealed that the MCF-7 breast cancer cells treated with DT-8 and VIN have almost similar profiles, especially when compared to those related to CDDP vs. K. As a matter of fact, among the discriminating metabolites in CDDP vs. K, the most abundant and significant are lactate and valine (FC = 1.59 and 1.24), and, also if not statistically significant, alanine (1.21), asparagines (2.24), aspartate (1.87), glutamate (1.11), hypoxanthine (1.69), isoleucine (1.11), lysine (1.24) and methionine (1.50), whereas phenylalanine and tyrosine (0.91 and 0.72, respectively) are present in smaller quantity. Formate and acetate characterized CDDP profiles, although with not significant value (FC = 0.34 and 0.38, respectively) showing a difference in the treatment between CDDP and VIN compared to DT-8. A common feature in all the three profiles was the low presence of lipids.

## 3. Discussion

The ^1^H-NMR-based metabolomic profiling of the treated MCF-7 cells accounts for the pharmacological effects of the tested drugs in cancer cells, whose metabolic phenotype encompasses alterations in glycolysis and the metabolism of amino acids, glycerophospholipids and nucleotides. It should allow us to get information about alterations of metabolic pathways underlying the proliferative capacity that cancer cells activate or change, and ultimately may support therapeutic strategies and drug discovery [19]. In this study, the discriminating comparison of the metabolites showed a high content of glucose and succinate fraction in DT-8 vs. K and VIN vs. K metabolic profiles. Typically, most of the glucose consumed by the cells is catabolized through glycolysis in pyruvate, which is transported into the mitochondria. In aerobic cell mitochondria, pyruvate supports the tricarboxylic acid cycle (TCA) and the electron transport chain (ETC), where oxidative phosphorylation occurs. Glucose catabolism coupled with oxidative phosphorylation has a high energy yield as ATP. In the most common metabolic phenotype, cancer cells convert much of the pyruvate into lactate, which, in DT-8 vs. K and VIN vs. K, shows a significant intracellular reduction, compared to CDDP vs. K [20]. The glucose catabolism into lactate has an extremely low energy yield; consequently, cancer cells require a high rate of glucose consumption to meet their energy and anabolic needs. Glucose-derived pyruvate not only supports the synthesis of acetyl-CoA, which is not only a precursor of synthesis of fatty acids, lipids and cholesterol, but also of the synthesis of non-essential amino acids such as aspartate and asparagine, through the activities of the pyruvate carboxylase and glutamate and oxaloacetate transaminases [21]. The ^1^H-NMR-based metabolomic profiling, although limited to cells lysates, highlights a possible significant glucose accumulation in the cells treated with DT-8 and VIN vs. K, and not with CDDP vs. K, but the reduced levels of aspartate and asparagine suggest that pyruvate should not be used as the substrate for their synthesis. In fact, even the accumulation of succinate, observed especially in DT-8 vs. K, supports this hypothesis, the presence of succinate could be involved in inhibiting the activity of PHD. Moreover, as recently reported, succinate is able to act as an intracellular messenger, inducing alterations in gene expression in tumors by targeting HIF-1α [22]. Another significant difference in the amino acid metabolites’ levels in the profiles of the samples DT-8 vs. K and VIN vs. K is represented by the glutamine. Glutamine is the most abundant amino acid in plasma; it can be metabolized into pyruvate and lactate through glutamate, α-ketoglutarate and through the citric acid cycle. This process, called glutaminolysis, is an additional important source of energy, carbon and nitrogen in cancer cells [23]. Under in-vitro cell culture conditions, glutamine is the second most consumed nutrient after glucose and its anaplerotic contribution has been shown to support the anabolic metabolism of cancer cells. Glutamine-derived glutamate has several fates in proliferating cells, including nucleic acid metabolism, glutathione (GSH) synthesis, and uridine diphosphate *N*-acetylglucosamine (UDP-GlcNAc) synthesis [21]. In our study, a small amount of intracellular glutamate was observed in DT-8 vs. K and in CDDP vs. K [24] compared to that observed in the profile of VIN vs. K in which there is little reduction. The amount of glutamate present in the cells after the DT-8 and CDDP treatment could be due to the export from the cell that is often associated with the import of cystine through the antiport with xCT [25]. The metabolism of glutamate after treatment could be linked to the need to detoxify the cells due to the pharmacological action induced by DT-8 and CDDP with respect to VIN. Indeed, GSH is synthesized from glutamate, glycine and cysteine. GSH oxidation by GSH peroxidase is coupled with the turnover of hydrogen peroxide (H_2_O_2_), a ROS by-product of mitochondrial oxidative phosphorylation. The oxidized glutathione (GSSG) is then reduced to GSH by the GSH reductase coupled with the NADPH oxidation. ROS turnover through this pathway requires a supply of NADPH, which can be generated from glucose by the pentose phosphate pathway [21]. A potential nutritional source in addition to glucose and glutamine is acetate. In fact, we observe a depletion of acetate in DT-8 vs K and in particular CDDP vs K compared to VIN vs K. Acetate, as has been recently reported, which represents a further origin of acetyl-CoA and, under conditions of metabolic stress such as low lipid availability and hypoxia, can become crucial in poorly vascularized regions in tumors [21]. Other metabolites identified in the metabolic profiles and showing similar behavior in DT-8 vs. K and VIN vs. K are hypoxanthine and methionine, which are present in lesser quantities than in the profiles of CDDP vs. K. Hypoxanthine is formed as a product of degradation of purine bases from nucleic acids. Intracellular concentrations of hypoxanthine are inversely related to the energy changes of adenylate and, therefore, to the energy currency of cellular ATP [26]. The essential amino acid methionine, identified as a discriminant metabolite in the profiles of DT-8 vs. K and VIN vs. K, falls within the pool of amino acids present in the least amount in the lysates. In addition to its role as a protein component, methionine connects to a number of important metabolic pathways that play key roles in nuclear functions (polyamines), detoxification (glutathione), cell membranes (phospholipids) and epigenetics (S-adenosylmethionine). Furthermore, the methionine cycle is intimately linked to folate metabolism and therefore it can indirectly modulate the biosynthesis of nucleotides. The MCF-7 cell line is dependent upon exogenous methionine intake, so at a first glance the metabolomic analysis, although limited to cell lysates, would suggest the depletion of this metabolite in the DT-8- and VIN-treated cells, and in contrast a significant uptake in CDDP vs. K samples. We might assume that there is a further cause for methionine depletion in DT-8 vs. K and VIN vs. K profiles. It is known that glycolysis is connected to the methionine cycle through the folate cycle. This pathway is important in cancer cells because the folate cycle supports both nucleotide biosynthesis and the synthesis of *S*-adenosylmethionine (SAM). SAM levels are influenced in particular by the folate cycle, important for the re-methylation of homocysteine in methionine, as well as for the synthesis of ATP. Both methionine and ATP are substrates for SAM formation. This creates a connection between the Warburg and the Hoffman effects [27]. Serine can be usually synthesized from glucose in sufficient amount through the intermediate 3-phosphoglycerate of glycolysis [28]. Increased demand for glycolysis by cancer cells for energy production can divert the flow of 3-phosphoglycerate from serine synthesis to glycolysis. The serine requirement for tumor cell proliferation can therefore link the effects of Warburg and Hoffman by connecting the glycolytic flow with the SAM synthesis. The reduction of methionine and ATP could result in an ineffective action of the SAM whose contribution is manifested by a specific cell cycle arrest followed by apoptosis. This arrest can be induced in mammalian and yeast cells by SAM limitation or methionine depletion. Cells show robust arrest in the G1 phase of the cell cycle due to the lack of stable pre-replication (pre-RC) complexes to initiate the S phase and a delay in G2/M is also observed [29]. The metabolic profiles resulting from DT8-treated cell lysates showed methionine depletion, which could, at least in part, explain the effect of the new biphenyl nicotinamide derivative DT8 (**3**) on cell cycle where it induced a blockade in the G1 phase at low doses and a delay in the G2/M phases. This behavior compared to that of VIN led to hypothesizing a multitarget action of DT-8. With regard to methionine depletion, as inferred from this metabolomic study, there is no substantial difference between DT-8 and VIN. The methionine uptake observed in the CDDP vs. K profile (Figure 5) is most likely due to its high affinity for heavy metal ions, including Pt(II). Inactivation of Pt ion normally occurs by interaction with glutathione, but CDDP cytoplasmic targets also include methionine [30]. The diverse metabolic response induced by CDDP in MCF-7 cells is also shown by the amount of glucose. Furthermore, CDDP is known to cause G2/M phase blockade in MCF-7 cells that are relatively resistant to CDDP treatment [31,32].

In the metabolic profiles obtained from the three cell models, we also observed the metabolites of the so called “one-carbon metabolism” (e.g., phenylalanine and formate), which contribute to numerous biosynthetic and cellular regulatory processes. Phenylalanine is an exogenous essential aromatic amino acid; it is converted into tyrosine, which in turn is metabolically degraded to acetoacetate and fumarate [33]. In all the profiles examined in this study, a reduction of both amino acids was observed. Formate is a product of folate metabolism, but it can be produced by other folate-independent processes, including the pathway of tryptophan degradation, the polyamine/methionine cycle and the methanol detoxification pathway [34]. Despite some apparent differences observed in the metabolomics of the three MCF-7-treated cell lysate samples with respect to controls, formate decrease (CDDP and DT-8 vs. K) or increase (VIN vs. K) are not statistically significant. The examination of the profiles of the discriminating metabolites, especially between treated samples (DT-8, VIN) and controls (K), shows a superimposable depletion of most essential and nonessential amino acids, which suggests the need to divert much glucose and glutamine to alternative metabolic processes to overcome treatment stress. In addition, the amino acids themselves play a role in biosynthesis, in reprogramming the metabolism in tumor cells as they can be used as sources of energy and help maintain the redox balance [35]. Another feature highlighted by the metabolic comparison between the drug-treated MCF-7 cells vs. controls (Figure 3) is the low lipid fraction, which prove that DT-8, VIN and CDDP are responsible for blocking their biosynthesis. The block of lipids’ synthesis could be the result of activations or inactivations of different but converging pathways. Glucose and glutamine, together with acetate, generate acetyl-CoA, which provides the demand for the synthesis of fatty acids and cholesterol for the growth of cancer cells [36]. Apparently, the lipid depletion in MCF-7 cell lysates, albeit observable for all three molecules, could be the result of targeting at different levels the expression of lipogenic genes, thus ultimately blocking tumor growth. Indeed, the lipid synthesis is mainly regulated by the transcription factor SREBP-1 (sterol regulatory element binding transcription factor 1), from which the genes ACLY (ATP citrate lyase), FASN (fatty acid synthase) and SCD-1 (stearoyl-CoA desaturase) are in turn regulated [37]. An overview of the ^1^H-NMR-metabolomic profiling, as shown by the comparative discriminant metabolite analysis between drug-treated MCF-7 cell lysates and respective untreated controls, indicates an important overlap of effects on the metabolites’ composition of breast cancer cells exerted by DT-8 and VIN, which are instead clearly separate from those caused by the treatment with CDDP. The amount of glucose and succinate in the profiles of DT-8 and VIN vs K reasonably suggest that the MCF-7 cells use these substrates for their metabolic change. The cell, most likely through the catabolism of glucose and glutamine, maintains the pool of different intermediates, which are used as building blocks for the assembly of various macromolecules, and the glycolytic intermediates leave the glycolysis to take part in different biosynthetic reactions. As a result, the enzymes that limit the speed within the branching pathways of glycolysis, which are frequently upregulated in tumors, act in intermediate processes and may become critical targets of drugs. The starting point of our metabolomic study was the possible mechanistic similarity between DT-8 and VIN, which is a well-established antimitotic alkaloid preventing the mitotic spindle formation and ultimately blocking cancer cell division [38]. The large overlap in the MCF-7 metabolic profiles of DT-8 and VIN, when compared with K, would suggest a similar mechanism of action but, as above observed, it is likely that DT-8 acts preferentially with respect to VIN, by mechanisms related to methionine depletion or *S*-adenosylmethionine (SAM) limitation, which, on the other hand, should be well associated with the cell cycle alterations observed with the DT-8 through a block in phase G1 at low doses [8]. The role in the control of the cell cycle of methionine depletion and/or SAM limitation may be linked to the control of the pre-replication complex (pre-RC) associated with the cell division cycle 6 (Cdc6) protein [29]. The formation of the pre-RC complex during the G1 phase is essential for chromosome replication to occur only once per cell cycle. The localization of the Cdc6 protein at the centrosome level for the formation of the pre-RC complex is crucial as it is the function of the centrosome, which is the center of organization of the microtubules. The formation of the pre-RC, being sensitive to the presence of methionine, dissociates from the DNA as the replication factor Cdc6 is downregulated [39]. Based on this reasonable hypothesis, congruent with the observed depletion of methionine, and previous findings, DT-8 and VIN interact with different targets, that is the pre-RC complex for the DT-8 and tubulin for VIN, both affecting the organization of the mitotic spindle during cell division. Nevertheless, further investigations, which may also include specific metabolomic studies on cell medium, could better buttress this hypothesis.

## 4. Materials and Methods

### 4.1. Cell Line and Treatment for Metabolomics Evaluation

The MCF-7 breast cancers cell line was obtained from the National Cancer Institute, Biological testing Branch (Frederick, MD, USA), and maintained in the logarithmic phase at 37 °C in 5% CO_2_ humidified air in RPMI 1640 medium supplemented with 10% fetal calf serum, 2 mM glutamine, penicillin (100 U/mL), and streptomycin (0.1 mg/mL).

The MCF-7 cells were seeded in exponential growth phase in culture flask of 75 cm^2^ (50,000 cells/mL), in the absence of serum. After 24 h, the cells were treated with aliquots of the drugs taken from 1 mM stock solutions of CDDP (H_2_O), DT-8 (DMSO) and VIN (DMSO), corresponding to IC_50_ (1.2 µM, 47 nM and 0.5 nM, respectively), while the controls were treated with vehicle solvent for 72 h to continuous treatment in complete medium. At 72 h, the adherent cells were washed, incubated with 0.2% trypsin/EDTA solution for 5 min, and collected by centrifugation (1200 rpm, 10 min). Cell pellets were washed three times with PBS, treated with cold methanol (300 µL), for cell lysis and, after solvent removal, by vortexing for 2 min and resting on ice for 15 min and, after solvent removal by gentle nitrogen flow, followed by vacuum, stored at −80 °C for further metabolomics evaluation.

### 4.2. Sample Preparation and NMR Measurements

For each sample, a volume of 600μL of saline buffer solution was added (in 100% D_2_O containing TSP as a chemical shift reference, δ = 0.00 ppm, NaN_3_ 2 mM, KH_2_PO_4_ 0.10 M, pH 7.4) and transferred in a 5 mm NMR tube [13,15]. 1D ^1^H one-dimensional ZGCPPR (water-suppression using composite pulses) and a CPMG (Carr–Purcell–Meiboom–Gill spin-echo sequence) experiment with a transverse-relaxation-filter incorporating pulse sequence and 2D ^1^H-^1^H J-resolved, ^1^H-^1^H COSY Correlation Spectroscopy, ^1^H-^13^C-HSQC Heteronuclear Single Quantum Correlation, and ^1^H-^13^C-HMBC Heteronuclear Multiple Bond Correlation spectra were recorded at 300 K on a Bruker Avance III NMR spectrometer (Bruker, Karlsruhe, Germany), operating at 600.13 MHz for^1^H observation, equipped with a TCI cryoprobe (Bruker, Biospin, Italy) incorporating a z axis gradient coil and automatic tuning-matching (ATM). ^1^H-NMR spectra were acquired with 128 transients, 16 dummy scans, a 5 s relaxation delay, an FID (free induction decay) size of 64 K data points, a spectral width of 12,019.230 Hz (20.0276 ppm), an acquisition time of 2.73 s, a total spin-spin relaxation delay of 1.2 ms (for CPMG experiments), and solvent signal saturation during the relaxation delay. The ^1^H-NMR spectra (ZGCPPR experiments), characterized by higher signal intensities and little affected by the presence of broad signals, were chosen for further processing using Topspin 3.5 (Bruker, Biospin, Italy) and Amix 3.9.13 (Bruker, Biospin, Italy), both for simultaneous visual inspection and the successive bucketing process. The entire NMR spectra (in the range 10.0–0.9 ppm) were segmented in fixed rectangular buckets of 0.005 ppm width and successively integrated. The spectral regions between 5.20–4.25, 3.70–3.60, 3.38–3.30, 3.18–3.07, 2.78–2.68, and 1.29–1.12 ppm, due to the residual peaks of solvents (water, methanol, ethanol, EDTA, DMSO), were discarded. The total sum normalization and the Pareto scaling procedure (performed by dividing the mean-centered data by the square root of the standard deviation) [40] were applied to minimize small differences due to sample concentration and/or experimental conditions among samples. Both explorative PCA (principal component analysis) and supervised OPLS-DA (Partial least squares and orthogonal partial least squares discriminant analyses were performed on samples [19,41] using SIMCA 14 software, (Sartorius Stedim Biotech, Umeå, Sweden). The validation of statistical models was performed and further evaluated by using the internal cross-validation default method (7-fold) and with the permutation test (20 permutations) available in SIMCA-P software (Sartorius Stedim Biotech, Umeå, Sweden) [41]. Moreover, a significant fold change ratio (FC, with probability of 0.05) for the discriminating metabolites among the observed samples was reported.

## 5. Conclusions

A ^1^H-NMR-based metabolomics analysis was performed to investigate the metabolic remodulation in MCF-7 cells treated with a new antiproliferative *N*-biphenyl nicotinamide (DT-8), as compared with two known drugs (CDDP and VIN). In total, 19 differential metabolites were defined for cell proliferation which requires greater use of nutrients (glucose and glutamine) and high flow through the biosynthetic pathways (nucleosides, amino acids). The comparative discriminant metabolite analysis between drug-treated MCF-7 cell lysates and respective untreated controls suggests that the effects on the cancer cell metabolomics of DT-8 and VIN are highly similar and distinct from those caused by the treatment with CDDP. One common feature of DT-8- and VIN-treated cancer cells is the amount of glucose, probably due to an accumulation and increased use of glutamine, lactate and methionine. On the other hand, DT-8 and VIN drugs could also downregulate glycolysis, leading to the intracellular accumulation of glucose and, consequently, to lower intracellular lactate levels. The results of PCA and OPLS-DA of the ^1^H-NMR spectra suggest the possibility that the cells try to overcome persistent stress by reprogramming the metabolism and activating alternative routes. One of these effects on MCF-7 metabolic responses for DT-8 is apparently related to methionine depletion, which could play a complex role, by blocking the organization of the mitotic spindle during cell division, than simply supporting protein synthesis. The effects of methionine imbalance after treatment with DT-8 and the disclosure of new biomolecular targets and mechanisms of action could allow the pharmacology of this new lead to be better understood and optimized by using the modern tools of the structure-based drug design.

## Figures and Tables

**Table 1:** Statistical parameters of OPLS-DA models for the classification of ^1^H-NMR experiments according to K (controls) and treated (CDDP, DT-8 and VIN). R^2^ is described by R^2^X and R^2^Y, which represent the fraction of variance of the X and Y matrix, respectively, Q^2^ = predictive capability.

OPLS-DA Parameters (1 + 1 + 0)	R^2^X	R^2^Y	Q^2^
K vs. CDDP	0.77	0.85	0.68
K vs. DT-8	0.89	0.99	0.98
K vs. VIN	0.82	0.98	0.90

**Table 2:** Discriminating metabolites (the first column reported the corresponding bucket for each metabolite in parenthesis), with FC ratio (and *p*-value < 0.05) obtained by multivariate analysis (MVA) for CDDP vs. K, DT-8 vs. K and VIN vs. K cell lysates.

	FC (*p*-Value)		
Metabolite (ppm)	CDDP vs. K	DT-8 vs. K	VIN vs. K
Acetate (1.9175)	0.38 (7.10 × 10^−2^)	0.88 (4.62 × 10^−1^)	1.36 (2.45 × 10^−1^)
Alanine (1.4775)	1.21 (4.59 × 10^−1^)	0.50 (5.28 × 10^−2^)	0.70 (4.74 × 10^−4^)
Asparagine (2.9375)	2.24 (7.35 × 10^−2^)	0.57 (9.51 × 10^−2^)	0.70 (1.16 × 10^−1^)
Aspartate (2.7975)	1.87 (7.18 × 10^−2^)	0.54 (2.02 × 10^−2^)	0.57 (4.11 × 10^−2^)
Formate (8.4625)	0.34 (1.45 × 10^−1^)	0.84 (8.03 × 10^−1^)	2.63 (2.62 × 10^−1^)
Glucose (5.2425)	0.62 (2.68 × 10^−2^)	3.85 (6.64 × 10^−7^)	2.84 (1.21 × 10^−6^)
Glutamate (2.3525)	1.11 (2.07 × 10^−1^)	1.05 (8.96 × 10^−1^)	0.93 (6.66 × 10^−1^)
Glutamine (2.4525)	0.97 (8.11 × 10^−1^)	0.61 (3.20 × 10^−2^)	0.66 (2.41 × 10^−3^)
Hypoxanthine (8.1975)	1.69 (7.00 × 10^−2^)	0.32 (6.88 × 10^−3^)	0.15 (6.73 × 10^−5^)
Isoleucine (0.9975)	1.11 (9.85 × 10^−2^)	0.70 (1.59 × 10^−3^)	0.83 (4.21 × 10^−3^)
Lactate (4.1325)	1.59 (1.53 × 10^−2^)	0.38 (1.42 × 10^−4^)	0.54 (1.55 × 10^−3^)
Leucine (1.7275)	0.96 (1.67 × 10^−1^)	0.73 (3.30 × 10^−5^)	0.77 (4.23 × 10^−3^)
Lysine (3.0325)	1.24 (1.57 × 10^−1^)	0.60 (5.07 × 10^−3^)	0.68 (2.08 × 10^−2^)
Methionine (2.6575)	1.50 (6.99 × 10^−2^)	0.41 (5.32 × 10^−3^)	0.50 (1.07 × 10^−2^)
*N*-acetyl glycoproteins (2.0675)	1.03 (4.03 × 10^−1^)	0.88 (4.69 × 10^−1^)	0.89 (2.45 × 10^−1^)
Phenylalanine (7.3375)	0.91 (6.50 × 10^−2^)	0.50 (3.53 × 10^−5^)	0.68 (2.37 × 10^−4^)
Succinate (2.4225)	0.96 (7.95 × 10^−1^)	1.42 (1.39 × 10^−1^)	1.20 (2.67 × 10^−1^)
Tyrosine (6.9025)	0.72 (1.68 × 10^−1^)	0.68 (7.11 × 10^−2^)	0.78 (1.97 × 10^−1^)
Valine (2.2675)	1.24 (4.33 × 10^−2^)	0.45 (4.90 × 10^−6^)	0.61 (7.84 × 10^−5^)

## Supplementary Materials

The following are available online, Figure S1: Representative one-dimensional 600 MHz ^1^H-NMR of ZGCPPR and CPMG spectra for lysate sample, Figure S2: Validation plot of 20 permutation tests for OPLS-DA model built for CDDP vs K (A) DT8 vs K (B) and (C) VIN vs K. Steep slope indicates well fit [2], Table S1: Chemical shifts (^1^H, ppm) and assignment of metabolite resonances identified in the 600 MHz ^1^H spectra of cell lysates (Letters in parentheses indicate the peak multiplicities; s, singlet; d, doublet; dd: doublet of doublet; t, triplet; q, quartet; m, multiplet; bs, broad signals), ^a^: AXP: can be adenosine mono-, di-, triphosphate; UDP-X can be UDP-glucose, UDP-galactose; UDP-Gluc-Nac [1], Table S2: Permutation test results of established OPLS-DA models: CDDP vs K (A) DT8 vs K (B) and (C) VIN vs K. Permutation test was used to check the validity of OPLS models. The intercept is a measure of the overfit [2]. e.
