# Supplementary material for: Novel Antiproliferative Biphenyl Nicotinamide: NMR Metabolomic Study of its Effect on the MCF-7 Cell in Comparison with Cisplatin and Vinblastine
Source: Molecules. 2020 Jul 31;25(15):3502. doi: 10.3390/molecules25153502 (PMC7435671; doi:10.3390/molecules25153502)
Supplement: Supplementary file 1 [file molecules-25-03502-s001.pdf]

molecules

Supplementary materials

Novel antiproliferative biphenyl nicotinamide: NMR metabolomic study of its effect on the MCF-7 cell in comparison with cisplatin and vinblastine

Laura Del Coco[1a], Maria Majellaro[2a], Angelina Boccarelli[3,*], Saverio Cellamare[2], Cosimo Damiano Altomare[2] and Francesco Paolo Fanizzi[1,*]

1 Department of Biological and Environmental Sciences and Technologies, University of Salento, Prov.le Lecce-Monteroni, I-73100 Lecce, Italy, laura.delcoco@unisalento.it (L.D.C.); fp.fanizzi@unisalento.it (F.P.F.)
2 Department of Pharmacy–Pharmaceutical Sciences, University of Bari, Via Orabona 4, 70125, Bari (Italy); ma.majellaro@gmail.com (M.M); saverio.cellamare@uniba.it (S.C.); cosimodamiano.altomare@uniba.it (C.D.A.
3 Department of Biomedical Sciences and Human Oncology, University of Bari, Piazza Giulio Cesare 11, 70124 Bari (Italy); angelina.boccarelli@uniba.it (A.B.)
* Correspondence: fp.fanizzi@unisalento.it (F.P. Fanizzi); angelina.boccarelli@uniba.it (A. Boccarelli)
a these authors contributed equally

Table S1. Chemical shifts (^{1}H, ppm) and assignment of metabolite resonances identified in the 600 MHz ^{1}H spectra of cell lysates (Letters in parentheses indicate the peak multiplicities; s, singlet; d, doublet; dd: doublet of doublet; t, triplet; q, quartet; m, multiplet; bs, broad signals). [a]: AXP: can be adenosine mono-, di-, triphosphate; UDP-X can be UDP-glucose, UDP-galactose; UDP-Gluc-Nac [1].

Metabolite	^{1}H (ppm)
Acetate	1.92 (s)
Alanine	1.48 (d), 3.79 (q)
Aspartate	2.67 (dd), 2.80 (dd)
AXP[a]	6.14 (d), 8.24 (s), 8.60 (s)
Asparagine	2.94 (dd), 2.86 (dd)
Choline	3.20 (s)
Formate	8.46 (s)
α,β–Glucose	3.40 (t), 3.71, 3.73, 3.88 (m), 4.64 (d), 5.22 (d)
Glycine	3.56 (s)
Glutamate	2.04 (m), 2.13 (m), 2.35 (m)
Glutamine	2.12 (m), 2.45 (m)
Hypoxanthine	8.19 (s), 8.20 (s)
Isoleucine	0.92 (t), 0.99 (d)
Lactate	1.32 (d), 4.11 (q)
Leucine	0.94 (d), 1.74 (m), 3.7 (m)
Lysine	1.72 (m), 3.02 (t)
Methionine	2.14 (s), 2.65 (t)
1-Methylhistidine	7.87, 7.08
Proline	2.00 (m), 2.05 (m), 2.34 (m), 3.32 (m), 4.12 (dd)

Phenylalanine	7.33 (d), 7.38 (m), 7.43 (m)
Threonine	1.32 (d), 4.26 (q)
Trypthophan	7.21 (d), 7.30 (d), 7.55 (d), 7.74 (d)
Tyrosine	6.89 (d), 7.19 (d)
UDP-X[a]	5.52, 5.62, 5.98 (bd), 7.88 (d)
Valine	1.00 (d), 1.04 (d), 2.26 (m)

Figure S1. Representative one-dimensional 600 MHz ^{1}H-NMR of ZGCPPR and CPMG spectra for lysate sample.

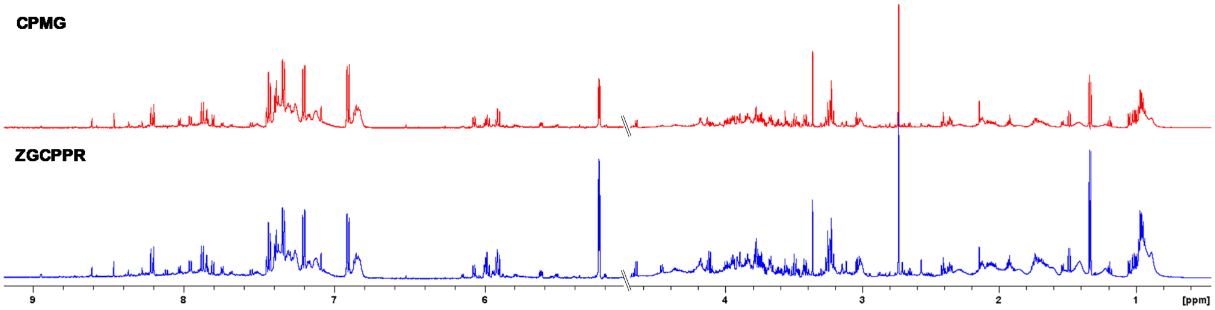

Figure S2. Validation plot of 20 permutation tests for OPLS-DA model built for CDDP vs K (A) DT-8 vs K (B) and (C) VIN vs K. Steep slope indicates well fit [2].

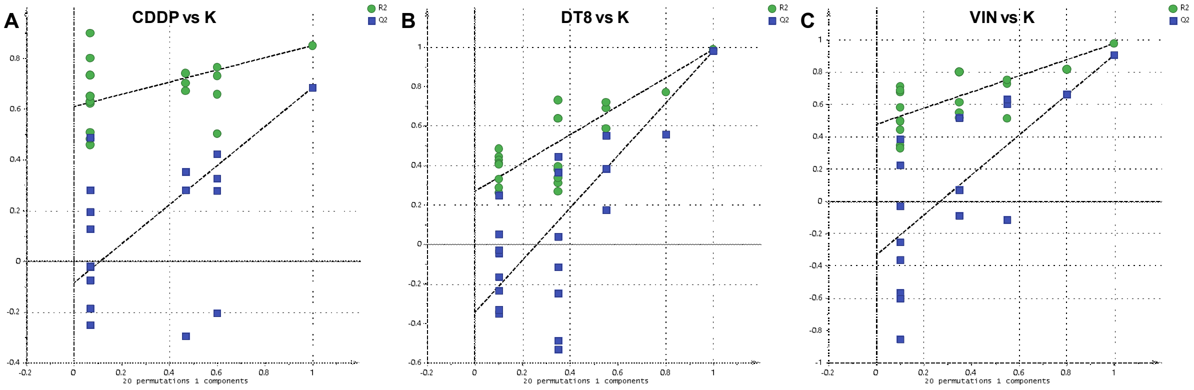

Table S2. Permutation test results of established OPLS-DA models: CDDP vs K (A) DT8 vs K (B) and (C) VIN vs K. Permutation test was used to check the validity of OPLS models. The intercept is a measure of the overfit [2].

**Intercept**	R^2	Q^2
**A**	0.581554	-0.13175
**B**	0.265203	-0.412201
**C**	0.347317	-0.604891

References

[1] Marks, V., Munoz, A., Rai, P., & Walls, J. D. (2016). ^{1}H-NMR studies distinguish the water soluble metabolomic profiles of untransformed and RAS-transformed cells. *PeerJ*, *4*, e2104.
[2] Eriksson, L.; Byrne, T.; Johansson, E.; Trygg, J.; Vikström, C. *Multi-and megavariate data analysis basic principles and applications*. Umetrics Academy: 2013.
